# The Interdisciplinary Challenge: A Case Report on Treating Recurrent Depressive Disorder Amidst Multiple Medical Comorbidities

**DOI:** 10.1192/j.eurpsy.2025.1258

**Published:** 2025-08-26

**Authors:** Z. C. G. Tan, I. C. S. Tan

**Affiliations:** 1 Department of Psychiatry and Behavioral Medicine, University of the Philiippines Philippine General Hospital, Manila, Philippines

## Abstract

**Introduction:**

This case highlights the interdisciplinary management of a 28-year-old Filipino female with recurrent depressive disorder and multiple medical comorbidities, including HIV, tuberculosis, and sexually transmitted infections. Managing depression in patients with complex medical conditions is especially challenging in resource-limited settings like the Philippines, where drug-to-drug interactions and access to high-quality care must be carefully navigated.

**Objectives:**

The objective of this case report is to illustrate the importance of personalized treatment decisions, the rationale for choosing Venlafaxine over SSRIs, and the value of psychodynamic psychotherapy in addressing underlying emotional conflicts in the context of maternal health challenges.

**Methods:**

The patient was referred to the Psychiatry service due to suicidal ideations. She has a history of depressive episodes linked to family stressors and initial flashbacks and nightmares related to her physical abuse from her previous partner. Her extensive comorbidities made the usual first-line antidepressants (SSRIs) unsuitable due to drug interactions. Venlafaxine 75 mg/day was selected, and psychodynamic psychotherapy, trauma-informed psychotherapy, and psychoeducation were integrated into her treatment. Coordination with infectious disease, obstetrics, and internal medicine specialists ensured comprehensive care.

**Results:**

Venlafaxine was effective in improving mood, sleep, and appetite, with no significant side effects. Regular follow-ups confirmed adherence to the medication, and psychodynamic psychotherapy helped her address deep-seated emotional conflicts, particularly surrounding her history of trauma. Ongoing psychiatric care, along with monitoring her medical conditions, provided a supportive framework for her continued improvement. Despite financial constraints, the patient adhered to her treatment regimen, showing improved mental health and commitment to her care.

**Conclusions:**

This case underscores the complexity of treating depression in patients with multiple comorbidities, particularly in resource-limited settings. The selection of Venlafaxine was crucial due to the potential interactions of SSRIs with the patient’s medical regimen. Psychodynamic and trauma-informed psychotherapy, combined with interdisciplinary collaboration, were key to the successful management of this case. This highlights the need for an integrated approach to mental health care, especially in low-resource environments, where interdisciplinary coordination is vital.

**Disclosure of Interest:**

None Declared

